# On the Efficacy of Handcrafted and Deep Features for Seed Image Classification

**DOI:** 10.3390/jimaging7090171

**Published:** 2021-08-31

**Authors:** Andrea Loddo, Cecilia Di Ruberto

**Affiliations:** Department of Mathematics and Computer Science, University of Cagliari, Via Ospedale 72, 09124 Cagliari, Italy; dirubert@unica.it

**Keywords:** image analysis, classification, deep learning, features extraction, seeds analysis

## Abstract

Computer vision techniques have become important in agriculture and plant sciences due to their wide variety of applications. In particular, the analysis of seeds can provide meaningful information on their evolution, the history of agriculture, the domestication of plants, and knowledge of diets in ancient times. This work aims to propose an exhaustive comparison of several different types of features in the context of multiclass seed classification, leveraging two public plant seeds data sets to classify their families or species. In detail, we studied possible optimisations of five traditional machine learning classifiers trained with seven different categories of handcrafted features. We also fine-tuned several well-known convolutional neural networks (CNNs) and the recently proposed SeedNet to determine whether and to what extent using their deep features may be advantageous over handcrafted features. The experimental results demonstrated that CNN features are appropriate to the task and representative of the multiclass scenario. In particular, SeedNet achieved a mean F-measure of 96%, at least. Nevertheless, several cases showed satisfactory performance from the handcrafted features to be considered a valid alternative. In detail, we found that the Ensemble strategy combined with all the handcrafted features can achieve 90.93% of mean F-measure, at least, with a considerably lower amount of times. We consider the obtained results an excellent preliminary step towards realising an automatic seeds recognition and classification framework.

## 1. Introduction

The last few decades have seen considerable growth in the use of image processing techniques to solve various problems in agriculture and the life sciences because of their wide variety of applications [1]. This growth is mainly due to the fact that computer vision techniques have been combined with deep learning techniques. The latter has offered promising results in various application fields, such as haematology [2], biology [3,4], or botany [5,6]. Deep learning algorithms differ from traditional machine learning (ML) methods in that they require little or no preprocessing of images and can infer an optimal representation of data from raw images without the need for prior feature selection, resulting in a more objective and less biased process. Moreover, the ability to investigate the structural details of biological components, such as organisms and their parts, can significantly influence biological research. According to Kamilaris et al. [7], image analysis is a significant field of research in agriculture for seeds, crops or leaves classification, anomaly or disease detection, and other related activities.

In the agricultural area, the cultivation of crops is based on seeds, mainly for food production. In particular, in this study, we focus on the field of plant science carpology, which examines seeds and fruits from a morphological and structural point of view. It generally has two main challenges: reconstructing the evolution of a particular plant species and recreating what the landscape was and, therefore, what its flora and fauna appeared. Professionals employed in this field typically capture images of seeds using a digital camera or flatbed scanner. Especially the latter provides quality and speed of workflow due to the constant illumination condition and the defined image size [8,9,10,11,12]. In this context, the seed image classification can play a fundamental role for manifold reasons, from crops, fruits and vegetables to disease recognition, or even to obtain specific feature information for archaeobotanical reasons, and so forth. One of the most-used tools by biologists is ImageJ [13,14,15]. It is defined as one of the standard types of image analysis software, as it is freely available, platform-independent, and easily applicable for biological researchers to quantify laboratory tests.

A traditional image analysis procedure uses a pipeline of four steps: preprocessing, segmentation, feature extraction, and classification, although deep learning workflows have emerged since the proposal of the convolutional neural network (CNN) AlexNet in 2012 [16]. CNNs do not follow the typical image analysis workflow because they can extract features independently without the need for feature descriptors or specific feature extraction techniques. In the traditional pipeline, image preprocessing techniques are used to prepare the image before analysing it to eliminate possible distortions or unnecessary data or highlight and enhance distinctive features for further processing. Next, the segmentation step divides the significant regions into sets of pixels with shared characteristics such as colour, intensity, or texture. The purpose of segmentation is to simplify and change the image representation into something more meaningful and easier to analyse. Extracting features from the regions of interest identified by segmentation is the next step. In particular, features can be based on shape, structure or colour [17,18]. The last step is classification, assigning a label to the objects using supervised or unsupervised machine learning approaches. Compared to manual analysis, the use of seed image analysis techniques brings several advantages to the process:

(i) It speeds up the analysis process;
(ii) It minimises distortions created by natural light and microscopes;
(iii) It automatically identifies specific features;
(iv) It automatically classifies families or genera.

In this work, we address the problem of multiclass classification of seed images from two different perspectives. First, we study possible optimisations of five traditional machine learning classifiers as adopted in our previous work [6], trained with seven different categories of handcrafted (HC) features extracted from seed images with our proposed ImageJ tool [19]. Second, we train several well-known convolutional neural networks and a new CNN, namely SeedNet, recently proposed in our previous work [6], in order to determine whether and to what extent a feature extraction performed from them may be advantageous over handcrafted features for training the same traditional machine learning methods depicted before. In particular, Table 1 depicts the study, contributions, and tools provided by our previous works and the one presented here.

The overall aim of the work is to propose a comprehensive comparison of seed classification systems, both based on handcrafted and deep features, to produce an accurate and efficient classification of heterogeneous seeds. It is important, for example, to obtain archaeobotanical information of seeds and to effectively recognise their types. More specifically, the classification addressed in this work is fine-grained, oriented to single seeds, rather than sets of seeds, as it is in [20]. In detail, our contribution is threefold:

(i) We exploit handcrafted, a combination of handcrafted, and CNN-extracted features;
(ii) We compare the classification results of five different models, trained with HC and CNN-extracted features;
(iii) We evaluate the classification results from a multiclass perspective to assess which type of descriptor may be most suitable for this task.

This research aims to classify individual seeds belonging to the same family or species from two different and heterogeneous seed data sets, where differences in colour, shape, and structure can be challenging to detect. We also want to highlight how traditional classification techniques trained with handcrafted features can outperform CNNs in training speed and achieve accuracy close to CNNs in this task.

The rest of the article is organised as follows. Section 2 presents state of the art in plant science work, with a focus on seed image analysis. Section 3 presents the data sets used and the classification experiments. The experimental evaluation is discussed in Section 4, and finally, in Section 5 we give the conclusions of the work.

## 2. Related Work

This work aims to classify seeds of different families, or species, according to the data set used. In general, computer vision techniques have been applied to this or similar tasks, even though no studies address heterogeneous seed identification or classification. For example, several authors have proposed methods to detect or classify types of seeds [5,6,21], leaves [22,23,24], and crops [25], to identify the quality of crops [26] or diseased leaves or crops [1,25,27,28,29], using both traditional and deep learning-based techniques. Table 2 gives a summary of the main methods and findings of the literature.

### 2.1. Leaf Detection and Classification

Several methods have been proposed for tasks similar to seed classification, such as leaf identification and recognition. Examples include a Support Vector Machine (SVM)-based method trained with leaf-related features such as shape, colour, and texture [22] and a mobile leaf detection system based on saliency computation to extract regions of interest, followed by segmentation with the region growing algorithm, which exploits both saliency map and colour features [24]. Finally, Hall et al. [23] use a combination of handcrafted and deep features as part of a classification system based on Random Forest (RF). It can classify the leaves of different plant species using a data set of over 1900 images divided into 32 species. The last method is particularly relevant and can be applied to seed images; however, it uses images of leaves acquired in ideal conditions, as well as Di Ruberto et al. [22], i.e., with an artificial background and not in actual conditions with a natural background, as in our investigation. As for Putzu et al. [24], the main focus is on the processing of leaves with complex artificial backgrounds in a mobile application scenario, which is far from the purposes of this work.

### 2.2. Leaf Diseases Identification

Works that are similar but oriented to the identification of diseases have been proposed by different authors [1,25,27,28,29]. Some examples include the work of Slado et al. [27], which used CaffeNet (a single-GPU version of AlexNet’s CNN) to identify leaf diseases; while AlexNet and GoogleNet have been used to identify 14 crop species and 26 different diseases [25]. In addition, LeNet has been used for diseased banana leaves recognition in [28]. Barman et al. [1] proposed a real-time citrus leaf disease detection and classification system based on the MobileNet CNN [30]. Finally, Gajjar et al. [29] proposed a novel CNN as part of a framework for real-time identification of diseases in a crop, tested on 20 different healthy and diseased leaves of four different plants. Each of the works in this section uses convolutional neural networks suitable for leaf disease detection. Contrary to the analysis carried out in our work, they did not study the handcrafted features that are particularly important in discriminating different types of seeds.

**Table 2:** Overview of existing works in this field with key insights from the proposed methods.

Work	Task	Main Method	Observations
[22]	Leaf detection	HC features + SVM	Ideal background
[23]	Leaf classification	HC+CNN features + RF	Ideal background
[24]	Leaf detection	saliency + colour features	Complex artificial
		+ SVM	background
[25]	Leaf disease	AlexNet and	No investigation
	detection	GoogLeNet	on HC features
[28]	”	LeNet	”
[27]	”	CaffeNet	”
[1]	”	MobileNet	”
[29]	”	Novel CNN	”
[31]	Crop detection	YOLOv3	Detection system
		modified	for monitoring
[26]	Crop quality	CNN features + SVM	No investigation
	detection		on HC features
[5]	Seed detection	HC features+LDA	Identification of single
			seed class
[21]	Seed germination	AlexNet modified	Identification of
	ability classification		single seed quality
[6]	Seed classification	HC features + ML methods	No optimisations
			on ML methods
		CNN methods	No investigation on deep
			features + ML methods

### 2.3. Classification of Crops

A pretty similar task to the one faced in this work is related to the classification of crops. For example, Junos et al. [31] proposed a detection system based on an improved version of YOLOv3 to detect loose palm fruits from images acquired under various natural conditions; on the other hand, Zhu et al. [26] realised a system to recognise the appearance quality of carrots, based on the SVM classifier trained with features extracted by CNNs. In particular, ResNet101 network offered excellent results. CNNs are also used in these works. In the first case, the task is different because the proposed system is a monitoring framework rather than a fine-grained classification system. In Zhu et al. [26], however, they employed features extracted from CNNs as in our work, although they did not employ handcrafted features as a comparison term for detecting the quality of carrots.

### 2.4. Seed Detection and Classification

The works most related to ours belong to this category. In particular, Sarigu et al. [5] performed a plum variety identification employing seed’s endocarp shape, colour, and texture descriptors followed by a Linear Discriminant Analysis (LDA) to obtain the most representative features, while Przybylo et al. [21] and Loddo et al. [6] employed CNNs. On the one hand, the first one used a modified version of AlexNet [32] and focused on the task of acorn germination ability classification based on the colour intensity of seed sections as a feature. On the other hand, a new CNN, called SeedNet, has been proposed by Loddo et al. [6] to classify and sort seeds belonging to different families or species. As in the first two works, we aim to classify seeds. Moreover, we investigated the same data sets employed in the latter work. The authors focused on a single seed variety in the first two works and employed only handcrafted features. As for our previous work [6], we proposed a new CNN for the classification and retrieval of fine-grained seeds without going into the details of handcrafted and deep features, which is one of the main purposes of the current work.

## 3. Materials and Methods

We leverage two data sets in this work. They contain images of heterogeneous seeds, both in number and in characteristics. They are publicly available on request. Each one was preprocessed as described in our previous work [6] and used for seed family or species classification using handcrafted or deep features. In the following, we start by describing the data sets in Section 3.1.1 and Section 3.1.2. We then provide the implementation details of the classification strategy. We validate the performance through an empirical evaluation with a 10-fold cross-validation strategy and visualise the process’s overall and class-specific discriminative features.

### 3.1. Data Sets Description

In this section, we describe the data sets used for the experiments.

#### 3.1.1. Canada Data Set

The Canada data set is publicly available [33]. It contains 587 images of seeds, organised into families. Every seed belongs to the *Magnoliophyta phylum*. Every image can have one of the following three different resolutions: 600×800, 600×480, and 600×400. We exploited this data set because

(i) It provides several different families, and
(ii) The background of the images is clean and requires a precise and unique preprocessing strategy.

**Figure 1:**
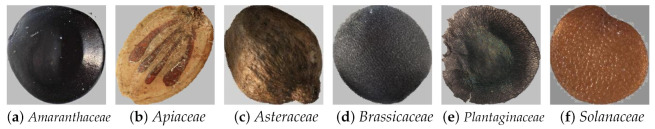
Samples of seed for each family present in the Canadian data set.

In particular, for the experiments, we selected the families considering the six most represented—*Amaranthaceae*, *Apiaceae*, *Asteraceae*, *Brassicaceae*, *Plantaginaceae*, and *Solanaceae* (23)—for a total of 215 seed images. Figure 1 shows a sample for each family, and Table 3 indicates the number of samples. Each original image contains a scale marker as a dimensional reference for the seed. It was removed following the preprocessing procedure proposed in [6].

#### 3.1.2. Cagliari Data Set

The basic collection of the *Banca del Germoplasma della Sardegna* (BG-SAR), University of Cagliari, Italy, was used to create the local data set. It consists of 3386 samples from 120 different plant species. Each seed is a member of the *Fabaceae* family and varies significantly in size and colour. The images have a resolution of 2125×2834 [34]. We used a preprocessing procedure defined by our previous work [6] to remove the background and extract single seeds for classification.

We chose the families with the most numerous samples, for a total of 23 different ones: *Amorpha*, *Anagyris*, *Anthyllis barba jovis*, *Anthyllis cytisoides*, *Astragalus glycyphyllos*, *Calicotome*, *Caragana*, *Ceratonia*, *Colutea*, *Cytisus purgans*, *Cytisus scoparius*, *Dorycnium pentaphyllum*, *Dorycnium rectum*, *Hedysarum coronarium*, *Lathyrus aphaca*, *Lathyrus ochrus*, *Medicago sativa*, *Melilotus officinalis*, *Pisum*, *Senna alexandrina*, *Spartium junceum*, and *Trifolium*, *Vicia faba*, for a total of 1988 seeds.

**Figure 2:**
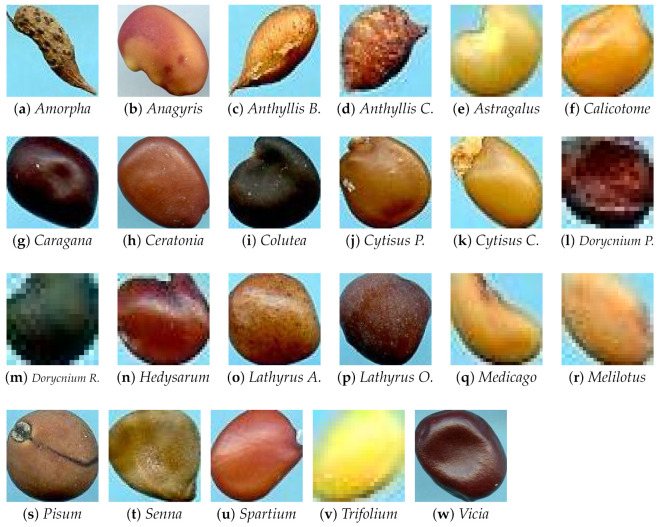
Samples of seed for each species present in the Cagliari data set.

Figure 2 depicts one sample from each family in the Cagliari data set, while Table 4 shows the number of samples.

### 3.2. Evaluation Metrics

The following metrics are used to evaluate the performance of each classification model: accuracy (Acc), precision (Pre), specificity (Spec), and recall (Rec). Accuracy is defined as the proportion of correctly labelled instances to the total number of instances. Precision is the proportion of true positives in a set of positive results. Specificity is the proportion of negative results that are correctly identified, and recall is the proportion of positive results that are correctly identified. They are defined as follows:

(1) $$Accuracy=\frac{TP+TN}{TP+TF+FP+FN},$$

(2) $$Precision=\frac{TP}{TP+FP},$$

(3) $$Specificity=\frac{TN}{FP+TN},$$

(4) $$Recall=\frac{TP}{TP+FN}.$$

TP, FP, TN, and FN indicate True Positives, False Positives, True Negatives, and False Negatives, respectively. Finally, since we are facing a multiclass imbalance problem, we also applied two of the most common global metrics for learning multiclass imbalance to evaluate the performance of the classifier [35]. The measures are the macro geometric mean (MAvG), defined as the geometric mean of the partial accuracy of each class, and the mean F-measure (MFM), which is the average of the F-measure computed for each class. They are defined as:

(5) $$MAvG={\left(\prod_{i=1}^{J}Acc_{i}\right)}^{\frac{1}{J}},$$

(6) $$MFM=\sum_{i=1}^{J}\frac{F-measure(i)}{J},$$

where *i* represents the current class and *J* the total number of classes. The F-measure(i) for class *i* is defined as:

(7) $$F-measure\left(i\right)=\frac{2\times TP(i)}{2\times TP(i)+FP(i)+FN(i)}.$$

We pinpoint that each of the metrics shown in this work has been calculated as a macro average of the number of classes.

### 3.3. Seed Classification

The handcrafted features were extracted using the ImageJ tool described in [19]. It can extract up to 64 different features. In particular, 32 are morphological shapes, 16 are textures, and 16 are colour intensities. Among the texture features, Haralick’s GLCM, which describes the arrangement of pixel pairs with the same grey level [36], was used to extract information of local similarities. They all permit their computation with the typical four different degrees: 0, 45, 90, 135. More precisely, we extracted the following second-order statistics from GLCM: energy, contrast, correlation, and homogeneity.

The handcrafted descriptors have been compared to deep features that were extracted from several different well-known network architectures: Vgg16, Vgg19, AlexNet, GoogLeNet, InceptionV3, ResNet101, Resnet18, Resnet50, and SeedNet. AlexNet [16], Vgg16 [37], and Vgg19 [38] are the shallowest among the tested architectures, being composed of 8, 16, and 19 layers, respectively. In all of these three cases, we extracted the features from the second last fully connected layer (fc7) for a total of 4096 features. GoogleNet [39], Inception-v3 [40], ResNet18, ResNet50, and ResNet101 [41] are much deeper, being composed of 100, 48, 18, 50, and 101 layers, respectively. In all of these cases, we extracted the features from the one fully connected layer for a total of 1000 features. Finally, SeedNet is a novel and lightweight CNN proposed in [6] for seed image classification and retrieval. We extracted the features from the last fully connected layer for a total of 23 features. The CNNs are known to have a sufficient representational power and generalisation ability to perform different visual recognition tasks [42]. Nevertheless, we fine-tuned the above CNNs on both data sets before the feature extraction in order to produce a fairer comparison to the standard machine learning classifiers trained with handcrafted features. In particular, we adopted the following classification strategy for both data sets:

(i) We split the data into 60% for training, 20% for validation, and 20% for test set;
(ii) We fine-tuned the CNNs on the training set, using the validation set to avoid overfitting;
(iii) We used 10-fold stratified cross-validation on training and validation set combined, in order to train the five classification algorithms;
(iv) We finally evaluated the classification performed on the test set.

The extracted features have been used as input to different classification algorithms in order to produce different classification models. The models considered are the following: k-Nearest Neighbors (kNN), Decision Tree (DT), Naive Bayes (NB), Ensemble (Ens), and Support Vector Machine (SVM). KNN uses the *k* nearest neighbour training examples in the data set as input, and a neighbour voting strategy ranks an object. Decision trees create a model that predicts the value of a target variable by learning simple decision rules inferred from the characteristics of the data. The deeper the tree, the more complex the decision rules and the more suitable the model. Naive Bayes classifiers are probabilistic models based on the application of the Bayes theorem with strong assumptions of independence between features. The Ensemble classifier is based on an ensemble of classifiers rather than a single one. The classifiers in the ensemble all predict the correct classification of each unseen instance, and their predictions are then combined using some form of voting system. Finally, SVM is a non-probabilistic binary linear classifier that assigns objects to a category, mapping the instances to points in space to maximise the width of the gap between categories.

To ensure the heterogeneity of the training set and keeping in mind that we faced a class imbalance problem, we trained each classifier with 10-fold stratified cross-validation to ensure that the proportion of positive and negative examples is respected in all folds in such a way that they contain a representative ratio of each class. For each case, we selected the model with the largest area under the ROC curve (AUC). The primary hyperparameters characterising each classifier were tuned in order to obtain a model with optimal performance. Furthermore, to make the results reproducible, we specify the values of the hyperparameters chosen for each model considered. For the kNN classifier, the distance metric adopted is the cityblock, and the number of nearest neighbours is 6, with a squared inverse distance weighting function. For the Decision Tree classifier, the maximum number of splits to control the depth of the trees is 50. The chosen distribution used to model the data is normal for the Naive Bayes classifier with a normal kernel smoother. For the Ensemble classifier, we chose the Adaptive Boosting (AdaBoost) method for multiclass classification. In particular, the learners are decision trees. Finally, for the SVM classifier, we used a polynomial kernel function of order 2, with an auto kernel scale parameter and a box constraint parameter equal to 1 to control the maximum penalty imposed on margin-violating observations and therefore to prevent overfitting. We evaluated the performance of each classifier using the same hyperparameters on both data sets.

## 4. Results and Discussion

**Figure 3:**
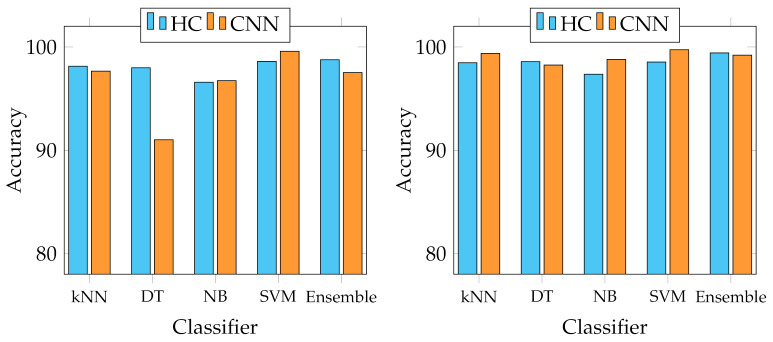
Accuracy trends with the different classifiers adopted. On the left, best accuracies obtained on Canada data set; on the right, on Cagliari data set.

**Figure 4:**
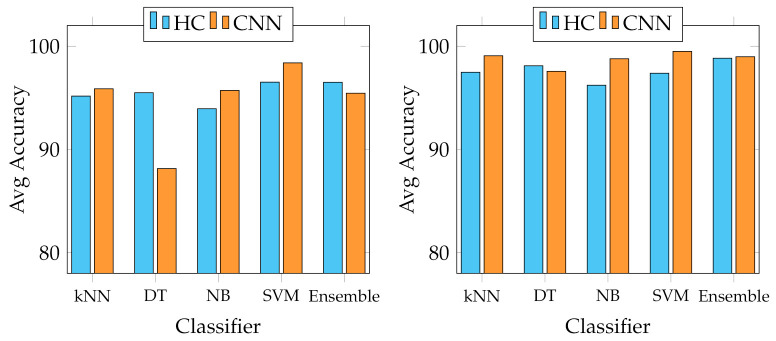
Average accuracy trends with the different classifiers adopted. On the left, average accuracies obtained on Canada data set; on the right, on Cagliari data set.

**Figure 5:**
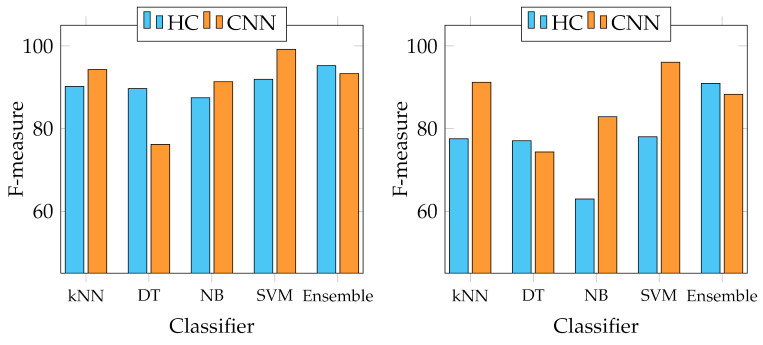
Best MFM trends with the different classifiers adopted. On the left, best MFM obtained on Canada data set; on the right, on Cagliari data set.

**Figure 6:**
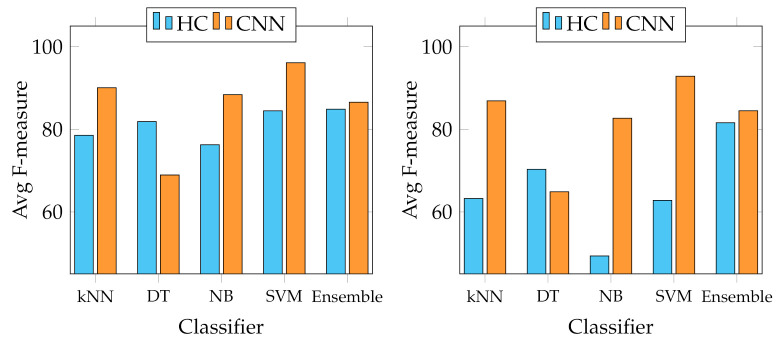
Average MFM trends with the different classifiers adopted. On the left, average MFM obtained on Canada data set; on the right, on Cagliari data set.

We report several results obtained from the experiments. First of all, four graphs are presented to show the general behaviour of the two sets of descriptors from two points of view. In fact, we report the best and average accuracies for both sets, as shown in Figure 3 and Figure 4. It works as a general indicator of the effectiveness of the features used for the task. In addition, we pinpoint their performance in the multiclass scenario. In particular, Figure 5 and Figure 6 show the behaviour of the MFM computed for the different classifiers. Secondly, in the Appendix A, we report Table A1–Table A5, in which the individual descriptor categories results obtained with each classifier are detailed.

The graphs in Figure 3 and Figure 4 show that each of the employed classifiers can achieve excellent classification accuracy on both data sets. However, from the results of the Canada data set, the Decision Tree classifier seems the least suitable for the task, especially when trained with CNNs descriptors, being the only one below 90% on average in that case. Although the other four strategies can achieve an accuracy above 90% in practically all cases, the Support Vector Machine seems the most appropriate in every experimental condition. It outperforms the others, averaging 98.38% and 99.49% on the Canada and Cagliari data sets, respectively, and a best of 99.58% and 99.73% with the features extracted from SeedNet on the Canada and Cagliari data sets, respectively. In general, and looking only at the accuracy, there seem to be no distinct performance differences in the two categories of descriptors to justify one over the other.

However, the scenario considerably changes when observing the results of the multiclass classification performance that we evaluated using the F-measure computed for all the classes, as indicated in Equation (6). In particular, Figure 5 shows that the best MFMs are generally lower than the accuracy on both data sets, even though the SVM reaches more than 99% of MFM on the Canada data set with ResNet50 and SeedNet descriptors and 96.07% with SeedNet-extracted features on Cagliari data set. In the last graph shown in Figure 6, the average performance obtained with the MFM again indicates the SVM trained with CNN descriptors as the most suitable choice for the task. Indeed, the SVM trained with CNN-extracted descriptors obtains 96.11% and 92.86% on Canada and Cagliari data sets, respectively.

As a general rule, on the one hand, the results provided with the extensive experiments conducted seem to show that the SVM trained with CNN-extracted features can accomplish the multiclass seeds classification task with performance that outperforms every other combination of descriptors and classifier analysed. Moreover, this solution seems to be robust, achieving the highest results in each comparative test. Those extracted from SeedNet performed excellently in all categories among the deep features, establishing themselves very well suited to the task. On the other hand, the results produced using the HC descriptors are satisfactory since they generally bring results comparable to the CNNs ones, even slightly lower than the best CNN descriptors case of the SVM. In general, the Ensemble strategy turns out to be the most appropriate when using HC descriptors, being able to reach 98.76% and 99.42% as the best accuracy and 95.24% and 90.93% as best the MFM on Canada and Cagliari data sets, respectively, and 96.50% 98.84% as the average accuracy and 84.88% and 81.59% as the MFM. Nevertheless, the different number of features that the two different categories have should also be considered. In particular, the handcrafted ones are 64 if combined, while the deep features are 4096 in the worst cases of AlexNet, Vgg16, and Vgg19, and 1000 in all the remaining CNNs. SeedNet is an exception because it has 23 features. Therefore, if we consider the number of discriminative features, the results obtained with the HC features are even more satisfactory and pave the way for possible combinations of heterogeneous features.

Since our investigation is the first attempt to study the problems of classifying fine-grained seed types with a large variety of different seed classes (up to 23), we leveraged known existing classification strategies that have been commonly used in other works closer to this [22,24,26,43,44]. Specifically, we employed kNN, Decision Tree, Naive Bayes, Ensemble classifier with AdaBoost method, and SVM. SVM is the most suitable for this task, probably due to its excellent capacity in distinguishing classes with closely related elements. This condition is evident in the Canada data set, containing seeds with heterogeneous shapes, colours, and textures. In contrast, the Cagliari data set is composed of similar classes, making the process more complex (see *Astragalus*, *Medicago*, and *Melitotus* as examples from Figure 2). For the same reasons, Decision Trees showed the most unsatisfactory results in this context because, with high probabilities, the features produced are insufficient to adequately represent all possible conditions of the internal nodes and realise an appropriate number of splits. Furthermore, as Figure 3 and Figure 4 show, the overall performance of the system in terms of accuracy indicates that the HC and CNN features are comparable, and in some cases, the first ones are better than the last ones. This behaviour is because, in general, both categories have high representational power for fine-grained seeds classification [6], both in this context and on the same data sets. However, the accuracy metric does not represent the detail of the multiclass issue faced in this work. For this reason, we adopted the mean F-measure in order to have a more unambiguous indication of the most suitable features for the task, keeping in mind the multiclass scenario.

**Figure 7:**
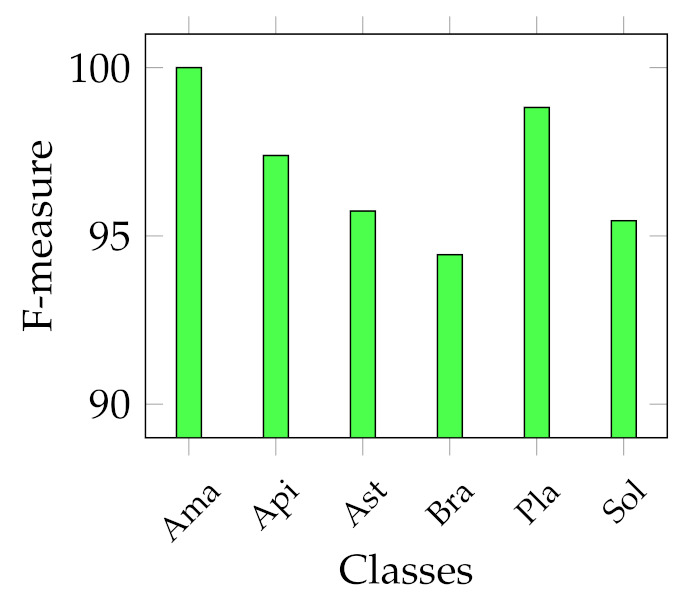
Classwise MFM on the best model for the Canada data set trained with deep features: SVM with ResNet50 features.

**Figure 8:**
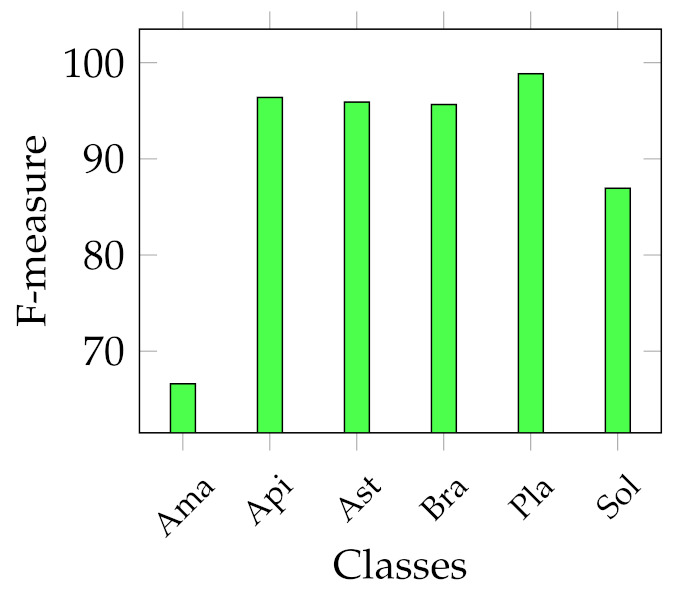
Classwise MFM on the best model for the Canada data set, trained with HC features: Ensemble with Texture features.

**Figure 9:**
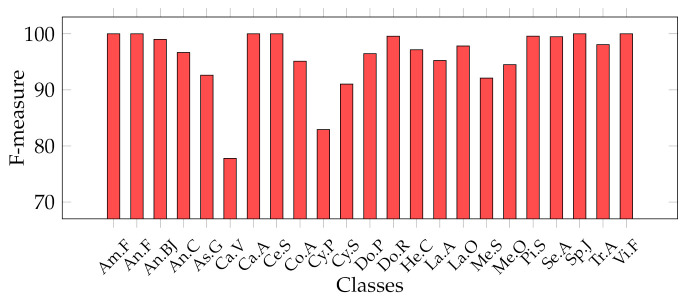
Classwise MFM on the best model for the Cagliari data set, trained with deep features: SVM with SeedNet features.

**Figure 10:**
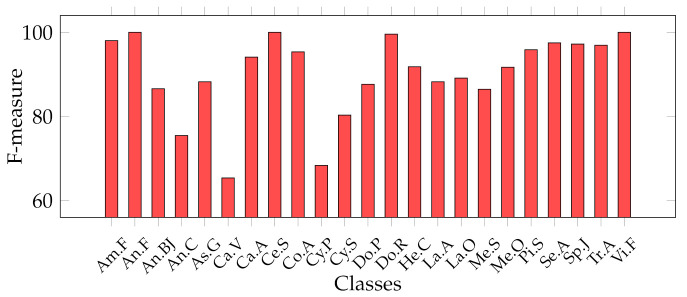
Classwise MFM on the best model for the Cagliari data set, trained with HC features: Ensemble trained with Shape + Texture + Colour (All) features.

Considering that we addressed a a multiclass classification problem, we provide Figure 7, Figure 8, Figure 9 and Figure 10, which represent the classwise MFM for each class of both features categories. In detail, regarding the Cagliari data set, Figure 9 and Figure 10 show that the most difficult classes are *Calicotome villosa* (with F-measures of 77.78% with the SeedNet features and 65.38% with all the HC features) and *Cystus purgans* (with F-measures of 82.93% with the SeedNet features and 68.35% with all the HC features), in both cases far below 90%. They are mostly misclassified with *Hedysarum coronarium* and *Cystus scoparius*, respectively. In both cases, this is certainly due to their similar shapes, and in the latter, certain seeds also have similar colours. As regards the Canada data set, Figure 8 shows that the *Amaranthaceae* (F-measure of 66%) class is mainly misclassified with *Solanaceae*, and vice versa, although to a lesser extent (F-measure of 86.95%). Even in this case, this is probably due to the similar shapes, but it is necessary to remark that the *Amaranthaceae* class contains only ten samples. The remaining four classes obtained an F-measure highly above 95%. On the other hand, Figure 7 represents the excellent representational power of the ResNet50-extracted features, considering that the F-measure of all the classes is above 95%, and, above all, the *Amaranthaceae* obtained 100%, overcoming the issues of the handcrafted features in discriminating it.

A final remark should be devoted to the execution time. We did not indicate the training time of the different CNNs employed because it is out of the scope of the work. However, we note that the training time was never less than 22 min on the Canada data set (AlexNet) and 21 min on the Cagliari data set (GoogLeNet) for the known architectures, while the SeedNet training lasted 4 and 12 min, respectively. Regarding the training time of the traditional classifiers, it was never above 1 min (the worst was Naive Bayes).

To sum up, the classification strategy based on the optimised SVM trained with SeedNet-extracted features is suitable for the seed-classification task, even in a multiclass scenario. This work shows how SeedNet is not only a robust solution for classification [6] but is also an outstanding feature extractor if coupled with the SVM classifier. The solution here obtained could also be more feasible than using SeedNet alone, considering the quicker training time of the SVM once provided with the selected features, in contrast to SeedNet alone.

While interesting results have been shown, our work suffers from some limitations. First, the best-performing solution relies entirely on one combination of descriptor and classifier, even though other categories of descriptors produced satisfactory results. Considering the properties of handcrafted features, combining them with deep features could improve the results, particularly in distinguishing the different classes of seeds more specifically. Second, every experimental condition assumed a preprocessing step before it, which needs to be tuned according to the data set employed. As a result, the trained classifier could have issues if applied to other data sets with different image conditions. Third, the training time of the best classification system strictly depends on the training time of the CNN adopted for the feature extraction. Efforts should be made in this sense in order to make a real-time system for the task addressed. Fourth, the dimensionality of the different feature vectors slightly changes if we compare handcrafted and deep descriptors. The first ones have a maximum of 64 features, while the second ones can have up to 4096. In this context, SeedNet is an excellent solution with only 23 features. A reasonable combination of heterogeneous descriptors could be made to investigate possible improvements, even followed by a feature reduction/selection. Fifth, as represented by the classwise performance, some classes are harder to distinguish because of their similar shapes and colours. In the case of Cagliari data sets, not even the deep features have overcome this issue. For this reason, the combination of heterogeneous descriptors could help recognise the most challenging classes.

## 5. Conclusions

In this work, we mainly focused on the problem of seed image classification. In this context, we specifically addressed an unbalanced multiclass task with two heterogeneous seed data sets, using both handcrafted and deep features. Based on shape, texture, and colour, the handcrafted features are general and dependent on the problem addressed, generating a feature vector with a maximum size of 64. Deep features were extracted from several known CNNs capable of performing different visual recognition tasks, generating a feature vector whose size is 1000 or 4096, except for SeedNet, which has 23. The features were then used to train five different classification algorithms, kNN, Decision Tree, Naive Bayes, SVM, and Ensemble. The experimental results show that the different feature categories perform best and comparably using SVM or Ensemble models for the Canada data set, with average accuracy values above 96.5%. The best model for the Cagliari data set is Ensemble for HC features and SVM for deep features. In both cases, the average accuracy values are above 99.4%. The MFM metric values give us essential information about how well the considered features can solve the unbalanced multiclass task. For both types of features, the Ensemble model achieves the best and comparable performance, with average values higher than 95.2% and 91%, respectively, for the data sets of Canada and Cagliari. When comparing HC- and CNN-based features, especially when considering the size of the feature vector, HC descriptors outperformed deep descriptors in some cases, as they achieved similar performance but with significant computational savings. In general, the results provided by the extensive experiments indicate that the SVM trained with the features extracted from the CNN can perform the task of multiclass seed classification with a performance that outperforms any other combination of descriptors and classifiers analysed. Moreover, this solution seems to be robust, obtaining the highest results in each comparative test. Among the deep features, those extracted by SeedNet performed excellently in all categories, establishing themselves as very well suited to the task and expressing SeedNet as a powerful tool for seed classification and feature extraction. It is also important to remark that the classwise performance highlighted that some classes are harder to distinguish because of their similar shapes and colours. For this reason, the combination of HC and deep descriptors could help recognise the most challenging classes.

In conclusion, SeedNet and CNNs, in general, have demonstrated their ability to offer convenient features for this task, achieving outstanding performance results with both data sets. However, if we consider the size of the feature vectors as a computational term, and the training time involved in the initial process, the HC feature performed satisfactorily, which is particularly desired for a real-time framework.

As a future direction, we aim to further improve the results obtained by investigating the possibility of combining the HC and CNN features, particularly to overcome the difficulties in recognising some seed classes and a feature selection step to reduce the dimensionality of the features. Finally, we also plan to realise a complete framework that can manage all the steps involved in this task, from image acquisition to seed classification, broadening our approach to distinguishing between seeds’ genera and species.

## Figures and Tables

**Table 1:** Contributions of current and our previous work in the context of seeds images analysis.

Work	Contributions
Loddo et al. [6]	- SeedNet CNN proposal
	- classification based on HC + ML methods vs. CNN
	- retrieval based on HC + ML methods vs. CNN
Loddo et al. [19]	- seed image acquisition and preprocessing schemes
	- open source ImageJ plugin for seeds feature extraction
	- open source ImageJ plugin for seeds classification
	- seeds classification with HC + ML methods
	- seeds classification with CNNs only
This work	- seeds classification with HC + ML methods
	- seeds classification with deep features + ML algorithms
	- study of the parameters optimisations in ML methods
	- comparison of the classification schemes for multiclass tasks

**Table 3:** Canada data set description: family name and number of samples.

Family	Samples
*Amaranthaceae (Ama)*	10
*Apiaceae (Api)*	56
*Asteraceae (Ast)*	49
*Brassicaceae (Bra)*	34
*Plantaginaceae (Pla)*	43
*Solanaceae (Sol)*	23

**Table 4:** Cagliari data set description: species name and number of samples.

Species	Samples	Species	Samples
*Amorpha fruticosa (Am.F)*	51	*Dorycnium rectum (Do.R)*	236
*Anagyris foetida (An.F)*	29	*Hedysarum coronarium (He.C)*	208
*Anthyllis barba jovis (An.BJ)*	51	*Lathyrus aphaca (La.A)*	52
*Anthyllis cytisoides (An.C)*	29	*Lathyrus ochrus (La.O)*	46
*Astragalus glycyphyllos (As.G)*	50	*Medicago sativa (Me.S)*	116
*Calicotome villosa (Ca.V)*	32	*Melilotus officinalis (Me.O)*	176
*Caragana arborescens (Ca.A)*	36	*Pisum sativum (Pi.S)*	121
*Ceratonia siliqua (Ce.S)*	45	*Senna alexandrina (Se.A)*	194
*Colutea arborescens (Co.A)*	42	*Spartium junceum (Sp.J)*	109
*Cytisus purgans (Cy.P)*	44	*Trifolium angustifolium (Tr.A)*	183
*Cytisus scoparius (Cy.S)*	65	*Vicia faba (Vi.F)*	31
*Dorycnium pentaphyllum (Do.P)*	42		

## Data Availability

All the features extracted, the models, and the material produced in this study are available at the following URL: GitHub repository.

## Abbreviations

The following abbreviations are used in this manuscript:

CNN	Convolutional Neural Network
HC	Handcrafted
RF	Random Forest
LDA	Linear Discriminant Analysis
GLCM	Gray Level Co-occurrence Matrix
TP	True Positive
TN	True Negative
FN	False Negative
FP	False Positive
Acc	Accuracy
Prec	Precision
Spec	Specificity
Rec	Recall
MAvG	Macro Average Geometric
MFM	Mean F-measure

## Appendix A. Numerical Results

This appendix section contains the numerical results of the experiments conducted, grouped by classifier. In particular, for every classifier we reported a table with its performance results. We employed kNN Table A1, Decision Tree Table A2, Naive Bayes Table A3, SVM Table A4, and Ensemble Table A5. More specifically, every table contains the detail of the individual HC descriptors of *Shape*, *Texture*, and *Colour* alone. Then, we inserted all the possible combinations: *Shape + Colour*, *Shape + Texture*, *Texture + Colour*, and *All*, which represent the combination of all the three categories. Then, the *Average HC* row represents the information used in Figure 4 and Figure 6. Regarding the CNN descriptors, every row represent the results obtained using that ConvNet as feature extractor. Finally, we reported the *Average CNN* row, which was also used in Figure 4 and Figure 6.

**Table A1:** Performance results obtained using HC descriptors and deep features with kNN classifier on Canada and Cagliari data sets. KNN were trained with k = 6 and cityblock as distance. DS indicates the data set.

DS	Descriptors	Acc	Prec	Spec	Rec	MAvG	MFM
Canada	Shape	94.88	80.07	96.86	82.66	78.86	81.13
	Texture	96.90	81.37	98.22	92.07	72.30	82.11
	Colour	86.20	48.94	91.58	51.00	40.91	49.18
	Shape + Colour	97.21	83.14	98.39	91.85	77.38	84.25
	Shape + Texture	98.14	88.56	98.92	94.69	86.25	90.19
	Texture + Colour	95.35	76.02	97.31	82.12	67.85	76.70
	All	97.52	85.23	98.56	89.52	81.56	86.20
	Average HC	95.17	77.62	97.12	83.42	72.16	78.54
	AlexNet	96.34	91.16	97.62	90.58	91.04	90.56
	Vgg16	94.01	82.45	96.19	85.57	82.04	83.52
	Vgg19	97.28	94.53	98.20	94.30	94.38	94.28
	GoogLeNet	94.97	89.64	96.82	90.07	88.88	88.80
	Inceptionv3	94.55	88.95	96.58	86.94	87.92	87.03
	ResNet101	95.81	90.82	97.28	88.91	90.64	89.65
	ResNet18	95.77	91.28	97.20	90.43	91.13	90.64
	ResNet50	96.42	92.80	97.62	92.19	92.66	92.35
	SeedNet	97.66	94.85	98.51	93.27	94.66	93.84
	Average CNN	95.87	90.72	97.34	90.25	90.37	90.07
Cagliari	Shape	95.43	39.32	97.56	45.07	5.59	41.07
	Texture	96.59	44.78	98.20	54.91	34.96	47.05
	Colour	98.24	71.93	99.07	76.72	66.01	72.75
	Shape + Colour	98.24	71.15	99.08	80.82	67.42	74.36
	Shape + Texture	97.04	53.67	98.44	63.48	42.65	55.95
	Texture + Colour	98.27	71.88	99.09	80.37	67.88	74.06
	All	98.47	74.70	99.20	83.79	70.90	77.53
	Average HC	97.47	61.06	98.66	69.31	50.77	63.25
	AlexNet	98.90	82.72	99.43	89.99	79.73	84.78
	Vgg16	99.03	84.23	99.49	88.91	82.99	86.02
	Vgg19	99.02	84.05	99.49	91.59	81.40	86.35
	GoogLeNet	99.23	87.78	99.60	93.59	85.70	89.55
	Inceptionv3	98.78	79.96	99.36	87.74	77.00	82.46
	ResNet101	98.80	81.17	99.37	87.78	76.49	82.88
	ResNet18	99.33	89.26	99.65	93.54	88.36	90.86
	ResNet50	99.15	86.25	99.55	91.01	84.56	87.86
	SeedNet	99.37	89.49	99.67	94.36	87.99	91.19
	Average CNN	99.07	84.99	99.51	90.95	82.69	86.88

**Table A2:** Performance results attained using HC descriptors and deep features by Decision Tree classifier on Canada and Cagliari data sets. Decision Tree were trained with 50 as maximum number of splits. DS indicates the data set.

DS	Descriptors	Acc	Prec	Spec	Rec	MAvG	MFM
Canada	Shape	93.02	74.12	95.72	73.53	72.44	73.80
	Texture	97.21	86.78	98.33	87.32	85.48	86.99
	Colour	89.15	59.42	93.44	58.83	53.57	58.91
	Shape + Colour	96.59	85.84	97.94	85.38	84.97	85.51
	Shape + Texture	97.21	89.94	98.28	89.53	89.71	89.68
	Texture + Colour	97.98	88.43	98.83	88.82	87.02	88.59
	All	97.36	89.75	98.39	89.63	89.50	89.68
	Average HC	95.50	82.04	97.28	81.86	80.38	81.88
	AlexNet	87.47	71.81	91.72	67.98	71.01	69.41
	Vgg16	91.01	71.69	94.23	70.58	69.30	70.74
	Vgg19	90.43	76.58	93.68	75.86	76.54	76.16
	GoogLeNet	88.99	67.14	92.87	74.19	65.13	69.21
	Inceptionv3	89.01	70.05	92.84	69.56	69.66	69.48
	ResNet101	83.74	60.97	89.27	62.65	60.01	61.71
	ResNet18	88.09	64.40	92.35	65.34	62.75	64.75
	ResNet50	87.03	69.08	91.47	68.17	68.62	68.16
	SeedNet	87.59	70.16	91.84	72.18	69.64	70.80
	Average CNN	88.15	69.10	92.25	69.61	68.07	68.94
Cagliari	Shape	97.45	63.29	98.65	67.89	55.55	63.47
	Texture	98.00	66.33	98.95	67.54	11.25	66.31
	Colour	97.25	57.82	98.55	60.71	48.11	58.34
	Shape + Colour	98.51	75.66	99.22	78.68	68.91	76.63
	Shape + Texture	98.43	73.43	99.18	76.09	65.88	73.95
	Texture + Colour	98.51	75.42	99.22	80.13	70.19	76.44
	All	98.58	76.33	99.25	79.00	70.46	77.06
	Average HC	98.10	69.75	99.00	72.86	55.76	70.31
	AlexNet	97.69	66.47	98.77	67.09	60.11	66.45
	Vgg16	98.25	73.34	99.07	76.53	71.03	74.33
	Vgg19	97.64	66.11	98.75	67.55	61.30	66.69
	GoogLeNet	97.85	68.66	98.87	68.31	64.17	68.33
	Inceptionv3	97.35	61.45	98.60	63.57	57.11	62.14
	ResNet101	96.63	52.77	98.21	54.59	8.45	53.09
	ResNet18	97.70	66.63	98.78	66.79	63.46	66.44
	ResNet50	97.27	60.49	98.55	60.41	55.13	60.18
	SeedNet	97.63	66.13	98.74	66.72	61.92	66.18
	Average CNN	97.56	64.67	98.70	65.73	55.85	64.87

**Table A3:** Performance results attained using HC descriptors and deep features by Naive Bayes classifier on Canada and Cagliari data sets. Naive Bayes were trained with kernel as distribution and normal as kernel type. DS indicates the data set.

DS	Descriptors	Acc	Prec	Spec	Rec	MAvG	MFM
Canada	Shape	93.02	71.80	95.79	73.97	67.29	72.24
	Colour	85.12	47.48	91.03	47.86	0.10	45.55
	Texture	96.12	83.07	97.67	86.20	81.07	84.07
	Shape + Colour	95.19	77.51	97.12	78.89	72.71	77.61
	Shape + Texture	96.59	86.24	97.91	90.64	85.23	87.45
	Texture + Colour	95.04	78.80	97.02	81.47	76.62	79.61
	All	96.59	85.85	97.92	90.75	84.84	87.32
	Average HC	93.95	75.82	96.35	78.54	66.84	76.26
	AlexNet	96.74	90.62	97.94	92.72	90.48	91.35
	Vgg16	92.26	81.50	95.08	80.43	80.93	80.20
	Vgg19	96.12	88.22	97.60	89.89	88.11	88.85
	GoogLeNet	96.74	90.62	97.94	92.72	90.48	91.35
	Inceptionv3	96.74	90.62	97.94	92.72	90.48	91.35
	ResNet101	95.66	86.31	97.30	90.24	86.06	87.88
	ResNet18	95.66	87.18	97.30	89.57	87.09	88.15
	ResNet50	95.66	86.28	97.29	89.22	86.15	87.47
	SeedNet	95.81	89.38	97.37	88.91	89.11	89.02
	Average CNN	95.71	87.86	97.31	89.60	87.65	88.40
Cagliari	Shape	95.93	45.89	97.85	50.57	7.17	45.88
	Texture	94.36	35.64	97.06	42.31	4.88	32.47
	Colour	97.36	65.72	98.61	65.45	59.72	62.95
	Shape + Colour	96.91	55.18	98.37	63.52	50.34	55.80
	Shape + Texture	96.10	43.95	97.94	54.91	36.74	46.04
	Texture + Colour	96.46	54.74	98.15	58.44	50.01	52.87
	All	96.41	47.67	98.11	59.18	41.31	49.39
	Average HC	96.22	49.83	98.01	56.34	35.74	49.34
	AlexNet	98.78	83.76	99.35	82.89	82.83	82.83
	Vgg16	98.77	83.71	99.35	82.53	82.75	82.72
	Vgg19	98.79	83.81	99.36	82.48	82.83	82.76
	GoogLeNet	98.78	83.94	99.35	82.68	82.91	82.85
	Inceptionv3	98.77	83.71	99.35	82.50	82.79	82.65
	ResNet101	98.77	83.40	99.35	82.48	82.45	82.46
	ResNet18	98.79	83.84	99.36	82.77	82.90	82.88
	ResNet50	98.76	83.68	99.34	82.18	82.71	82.46
	SeedNet	98.78	83.68	99.35	82.75	82.68	82.71
	Average CNN	98.78	83.73	99.35	82.58	82.76	82.70

**Table A4:** Performance results attained using HC descriptors and deep features by SVM classifier on Canada and Cagliari data sets. SVM was trained with a polynomial kernel of order 2. DS indicates the data set.

DS	Descriptors	Acc	Prec	Spec	Rec	MAvG	MFM
Canada	Shape	94.26	77.32	96.49	77.94	75.64	77.56
	Colour	92.87	69.54	95.80	69.36	61.89	68.89
	Texture	97.52	88.31	98.49	87.98	87.09	88.13
	Shape + Colour	96.74	83.51	98.08	83.72	81.24	83.54
	Shape + Texture	97.98	90.85	98.77	92.51	90.26	91.50
	Texture + Colour	98.60	91.89	99.19	91.99	91.19	91.92
	All	97.67	89.44	98.60	90.29	88.80	89.78
	Average HC	96.52	84.41	97.92	84.83	82.30	84.47
	AlexNet	98.94	97.77	99.32	97.54	97.74	97.57
	Vgg16	95.72	87.94	97.22	89.50	87.82	88.55
	Vgg19	98.75	97.60	99.16	97.34	97.57	97.45
	GoogLeNet	98.74	97.54	99.15	96.05	97.52	96.73
	Inceptionv3	97.25	94.16	98.23	92.69	93.97	93.24
	ResNet101	97.70	95.11	98.48	95.03	95.07	95.06
	ResNet18	99.15	98.40	99.42	98.01	98.39	98.18
	ResNet50	99.58	99.23	99.71	99.12	99.22	99.17
	SeedNet	99.58	99.07	99.73	99.08	99.05	99.06
	Average CNN	98.38	96.31	98.94	96.04	96.26	96.11
Cagliari	Shape	94.60	31.25	97.11	37.06	22.99	33.13
	Texture	96.53	48.07	98.16	50.46	42.24	48.92
	Colour	98.51	76.47	99.21	79.79	74.36	77.48
	Shape + Colour	98.32	72.91	99.12	79.73	68.85	75.18
	Shape + Texture	96.68	47.97	98.25	54.33	39.48	50.06
	Texture + Colour	98.54	76.79	99.23	80.14	74.21	78.01
	All	98.49	73.97	99.21	81.18	70.01	76.74
	Average HC	97.38	61.06	98.61	66.10	56.02	62.79
	AlexNet	99.48	91.96	99.73	94.62	91.31	92.99
	Vgg16	99.27	88.24	99.62	90.49	87.29	89.14
	Vgg19	99.54	93.08	99.75	94.55	92.71	93.73
	GoogLeNet	99.55	93.08	99.76	94.16	92.52	93.52
	Inceptionv3	99.40	90.81	99.68	92.31	90.29	91.47
	ResNet101	99.25	88.64	99.61	91.48	86.93	89.62
	ResNet18	99.67	95.08	99.83	96.82	94.80	95.83
	ResNet50	99.53	92.94	99.75	93.94	92.43	93.34
	SeedNet	99.73	95.47	99.86	96.91	95.11	96.07
	Average CNN	99.49	92.14	99.73	93.92	91.49	92.86

**Table A5:** Performance results attained using HC descriptors and deep features by Ensemble classifier on Canada and Cagliari data sets. The ensemble was trained with a bag method and tree as a learner. DS indicates the data set.

DS	Descriptors	Acc	Prec	Spec	Rec	MAvG	MFM
Canada	Shape	95.04	77.84	97.02	80.53	74.78	78.87
	Texture	98.76	94.28	99.25	96.64	94.01	95.24
	Colour	89.92	59.61	93.93	62.59	53.60	60.34
	Shape + Colour	97.36	86.73	98.45	87.98	85.31	87.21
	Shape + Texture	98.14	90.49	98.90	93.00	89.77	91.50
	Texture + Colour	98.29	90.08	99.01	91.58	88.80	90.67
	All	97.98	89.25	98.80	92.18	87.97	90.31
	Average HC	96.50	84.04	97.91	86.36	82.03	84.88
	AlexNet	96.28	89.31	97.70	88.85	88.87	88.59
	Vgg16	93.64	76.20	96.13	79.52	75.03	77.32
	Vgg19	97.52	92.75	98.47	94.04	92.64	93.30
	GoogLeNet	94.73	83.92	96.76	85.62	83.64	84.68
	Inceptionv3	95.19	84.70	97.13	86.80	83.98	85.12
	ResNet101	93.95	81.66	96.31	85.07	81.29	82.84
	ResNet18	95.81	88.52	97.39	90.18	88.30	89.10
	ResNet50	96.59	91.46	97.84	90.14	91.35	90.69
	SeedNet	95.35	87.38	97.10	87.51	87.09	87.40
	Average CNN	95.45	86.21	97.20	87.53	85.80	86.56
Cagliari	Shape	98.15	70.75	99.03	76.62	65.90	72.61
	Texture	98.56	72.96	99.25	77.29	66.68	73.96
	Colour	98.27	73.13	99.09	76.88	70.08	74.37
	Shape + Colour	99.29	88.35	99.63	91.68	87.01	89.55
	Shape + Texture	99.02	81.53	99.49	86.78	77.19	82.95
	Texture + Colour	99.15	85.14	99.56	89.20	83.67	86.74
	All	99.42	89.54	99.70	93.53	88.13	90.93
	Average HC	98.84	80.20	99.39	84.57	76.95	81.59
	AlexNet	99.02	82.69	99.49	91.15	79.29	85.05
	Vgg16	99.06	84.87	99.50	87.16	83.79	85.73
	Vgg19	99.07	83.55	99.51	91.11	80.73	85.89
	GoogLeNet	99.16	85.39	99.56	90.67	83.23	87.34
	Inceptionv3	98.81	79.11	99.38	88.60	75.89	82.20
	ResNet101	98.40	72.45	99.17	83.57	63.71	74.96
	ResNet18	99.20	86.39	99.58	91.52	83.97	88.28
	ResNet50	98.93	80.64	99.44	88.23	77.28	83.20
	SeedNet	99.21	85.58	99.59	92.22	83.08	87.63
	Average CNN	98.98	82.30	99.47	89.36	79.00	84.48
